# Exploring Conceptualizations of COVID-19 Risk in Ideologically Distinct Online Communities: A Computational Grounded Theory Analysis

**DOI:** 10.2196/67968

**Published:** 2025-06-24

**Authors:** Tiwaladeoluwa B Adekunle, Jeremy Foote, Toluwani E Adekunle, Nathan TeBlunthuis, Laura K Nelson

**Affiliations:** 1 Feinberg School of Medicine Northwestern University Chicago, IL United States; 2 Purdue University West Lafayette, IN United States; 3 Calvin University Grand Rapids, MI United States; 4 University of Texas Austin, TX United States; 5 University of British Columbia Vancouver, BC Canada

**Keywords:** risk perception, online communities, computational methods, topic modeling, word embeddings, COVID-19, SARS-CoV-2, coronavirus, pandemic, grounded theory, conceptualizations, computational grounded theory, comment, thematic analysis, qualitative

## Abstract

**Background:**

The COVID-19 pandemic has had a profound impact on societies and economies around the globe, and experts warn about the potential for similar crises in the future. Risk communication theories underscore that while the potential for harm is objective, risk perception is a subjective, socially derived interpretation. While there is broad literature on the social construction of risk, fewer studies examine the role of communities—online or offline—in developing and reinforcing distinct interpretations of the same risk event. During COVID-19, online communities emerged as individuals sought to make sense of the ongoing crisis. These communities offer an opportunity to gain important insights into how concerned public collectively interprets risk and create group identities, informing public health strategies.

**Objective:**

This study aims to, first, explore how online communities with distinct ideologies create and reinforce divergent conceptualizations of risk and, second, identify the role of group identity in shaping the development and communication of risk interpretations in these communities.

**Methods:**

We used computational grounded theory, a multistep approach that includes pattern detection, hypothesis testing, and pattern confirmation to explore interpretations of risk and group identity in about 500,000 comments from the subreddits r/LockdownSkepticism and r/Masks4All. In the pattern detection step of this study, we grouped comments by the post they were made on and then used latent Dirichlet allocation topic modeling to identify 10 topics based on the frequency of term co-occurrence. In the hypothesis refinement step, we conducted a qualitative thematic analysis of 30 posts under each topic using Braun and Clarke’s approach. Finally, in the pattern confirmation step, we trained a Word2Vec word embedding model to validate emerging themes from the second step.

**Results:**

This study found that Masks4All and LockdownSkepticism both centered risk in their conversations, but with divergent concerns related to the threat of COVID-19. While Masks4All emphasized the threat to health, LockdownSkepticism questioned the necessity of preventive measures and focused on other risks: the threat to the economy, educational disruptions, and social isolation. Group identity was also found to shape collective meanings around risk, as community members in both subreddits affirmed group positions and condemned the outgroup.

**Conclusions:**

This study demonstrated that while both communities were concerned about COVID-19, their perceptions of risk focused on different aspects of the same risk event. This underscores the need for targeted interventions that engage with divergent ideologies and value systems across groups of people.

## Introduction

### Overview

Crisis events such as the COVID-19 pandemic create informational voids, which require interpretation and human action in the absence of robust information and evidence [1,2]. While the potential for harm is an objective consequence of threats such as the COVID-19 pandemic, risk is a social construct that emerges from communication within and between individuals [3,4]; how risky an individual perceives an event or a behavior to be is contingent upon a variety of factors, including values, ideologies, and identities [5,6]. Groups of people can form and reinforce collective perceptions of risk [4]. To better understand how concerned publics make sense of a risk event critical to public health, we compared divergent conceptualizations of COVID-19 risk in 2 ideologically distinct online communities on Reddit: Masks4All (M4A) and LockdownSkepticism (LS). Specifically, this paper explores (1) the cocreation of shared risk interpretations and (2) the role of group identity in the development and communication of risk meanings online.

### Social Creation of Risk

#### Overview

The Social Amplification of Risk framework (SARF) describes the social processes that shape experiences and consequences of risk [4]. Through social and communicative processes, risk can be amplified or attenuated, such that a group’s perceptions significantly differ from technical estimates by experts. Amplification channels include informal social networks, and amplification stations might include opinion leaders, cultural and social groups, government agencies, public relations information offices, and news media [4]. SARF has been used to help explain public attitudes and behavior during public health events, including the COVID-19 pandemic [7,8].

Political ideologies can shape how people perceive crisis events. For example, conservatives and liberals have been found to have different domains of risk perception, with liberals perceiving hazards that impact the collective (such as climate change) as “riskier” than conservatives [9]. Another study found that while liberals and conservatives had similar perceptions of COVID-19 risk, liberals were more willing to take precautions than conservatives [9]. Nonpolitical values have also been found to impact individuals’ perceptions of risk. For example, people with stronger egoistic values are more willing to accept nuclear energy [10].

SARF, combined with research on the role of pre-existing values, provides mechanisms to explain how groups can come to hold conceptions of risk which are similar within the group but differ from the risk perceptions of other groups. scholars have argued for the importance of tailoring health messaging to the concerns of specific groups [11] and for understanding the risk perceptions of different publics. However, while the constitution of these publics and the underlying communicative processes through which their concerns take shape have rich theoretical support, rarely have these publics been studied empirically [12], likely due to the difficulty and cost of identifying and gathering data from distinct large-scale groups.

#### Risk Creation in Online Communities

Online communities provide a novel opportunity to study the social creation of risk in times of crisis, both because they are an increasingly important source of critical information and support [13,14] and also because they provide large-scale, in situ data that can be used to understand how groups cocreate perceptions of risk. Individuals engage in online communities to navigate health concerns [15]. Online interaction was particularly critical during the COVID-19 pandemic, due to the mitigation strategies that included social distancing and isolation [16]. These communities emerged as spaces for engagement and cocreation of meaning during the COVID-19 crisis, and online discussions were found to contribute COVID-19 risk interpretations [16].

Online communities also provide a unique setting to study the role of group identity. While many community members passively consume content, other members of online communities feel a deep attachment to their communities and strongly identify with the group [17]. Social Identity Theory (SIT) posits that groups engage in bias toward members of groups that they identify with. Social identity, when salient, has been theorized to impact perceptions of risk [18].

Drawing on SIT and SARF, the research questions (RQs) were posed as (1) RQ1: What are the differences and similarities in constructions of COVID-19 risk in cautious (r/Masks4All) and skeptical (r/LockdownSkepticism) communities? (2) RQ2: How does group identity relate to the coconstruction of risk in these communities?

To address each of these RQs, we applied computational grounded theory, a 3-step approach (pattern detection, hypothesis refinement, and pattern confirmation) developed by Nelson (2020) [19].

## Methods

### Ethical Considerations

This study uses publicly available social media data; as such, we did not seek human participants’ ethical review. To limit the potential of author identification, we paraphrased all quotes included in this manuscript.

### Research Design Overview

To compare conceptualizations of COVID-19 risk in 2 communities with profoundly different perceptions of COVID-19, we identified communities that were discussing COVID-19–related risks on Reddit and chose one very cautious community (r/Masks4All) and one community skeptical of health concerns (r/LockdownSkepticism). We then collected all of the comments (about 500,000) posted in these 2 communities in the year 2020. This data was retrieved using the Pushshift application programming interface [20].

Reddit is a commonly used platform for studying online communication, including communication about public health [21,22]. Reddit is composed of hundreds of thousands of active communities known as subreddits, where individuals coalesce around issues of shared interests. Reddit data are frequently used in communication research, facilitating the study of communicative processes with implications for online and offline realities [23-25].

Data collected in 2020 was ideal for this study because this was at the onset of the COVID-19 pandemic, where individuals and communities developed interpretations of a new danger in communication with one another. New hazards lead to the formulation of mental representations shared by subcultures [26]. Examining these 2 subreddits at the beginning of the pandemic thus allowed for unique insights into subjective meaning-making within and across these distinct communities.

The analytic process for this study is derived from Nelson’s computational grounded theory [19]. Computational grounded theory is a methodological framework that combines the strengths of computer-assisted text analysis with those of the human interpretation central to traditional qualitative research. The 3 steps included in this approach include pattern detection, hypothesis refinement, and pattern confirmation. Other researchers have previously applied computational grounded theory to analyze communication in other subreddits [27].

#### Pattern Detection

In the pattern detection step of this study, we combined comments from both communities and grouped comments by the post on which they were made. We then used Latent Dirichlet Allocation (LDA) [28] topic modeling to identify common topics discussed. LDA topic modeling identifies topics based on how often words occur together in a corpus [28]. It requires researchers to specify the number of topics; we ran a coherence analysis to identify the optimal number of topics, and members of the research team then reviewed the top words associated with each topic to ensure that topics were distinct. For each topic identified by LDA, we retrieved the conversations and aggregated comments from the top 30 posts associated with that topic for each subreddit. By training the same LDA model for both communities, we can identify conversations across communities that are about similar concepts, allowing us to efficiently identify the similar and contrasting ways that each community discusses these concepts. Instead of using LDA topics as themes, we used topic modeling to direct our attention in a way that would ensure that we captured the diversity that exists in the types of conversations that were occurring in these communities.

#### Hypothesis Refinement

Next, we conducted a qualitative thematic analysis of the comments under each topic for each subreddit. We did a deep reading of the comments, paying special attention to whether or how these comments translate into statements of position and identity for each subreddit. During this step, we also critically assessed the validity of computational output, based on the patterns identified from our qualitative analysis [19].

We followed Braun and Clarke’s [29,30] method for thematic analysis. For each subreddit, members of the research team read through the dataset and made note of their thoughts and impressions. Next, we began the process of open coding, identifying and categorizing all aspects of the data that were relevant to our research questions. Based on these codes, we collaboratively interpreted themes by looking for patterns within and between the codes. Finally, we compared the themes identified in each subreddit.

#### Pattern Confirmation

In this step, we identified the key findings from the refined hypothesis step, which were amenable to computational validation and exploration. We trained a Word2Vec word embedding model for each community, and used these models to validate differences in how concepts are situated differently for each community. The gensim library [31] was used for both topic modeling and word embedding.

### Research Team

The research team is composed of PhD-trained social scientists with expertise in Communication, Public Health, and Sociology. Members of the research team have expertise in computational social science, qualitative methods, and computational grounded theory. The 2 coders for the qualitative portions of this study are qualitative research experts.

## Results

### Identification of Topic

The coherence metrics suggested that 10 topics were appropriate for an LDA model of this data. We labeled each topic based on the content of comments within them. Multimedia Appendix 1 includes each topic name and a summary of the type of comments most associated with that topic.

### Thematic Analysis

The thematic analysis was conducted on the 30 conversations most associated with each LDA topic for each subreddit. Following best practices, all direct quotes included in this section have been paraphrased using a combination of manual alterations and artificial intelligence (ChatGPT) to minimize the potential of individual identification of community members [32]. Guided by our research questions, we describe and elaborate on the salient subthemes that emerged related to differences and similarities in constructions of COVID-19 risk and the cocreation of group identity.

### Differences and Similarities in Constructions of COVID-19 Risk

#### Conceptualization of Hazard Severity

In the LS subreddit, contributors minimized the severity of COVID-19 by questioning the dangers associated with infection and arguing that there was nothing uniquely damaging about the virus. As one user shared,

We're in a pandemic...’ Seriously? I mean, yeah, technically we are, but if folks like you didn't keep throwing ‘COVID-19’ in everyone's faces, it would feel just like any other year.

In addition, users speculated about possible explanations for specific COVID-19 symptoms and the increased rates of illness and mortality. As one user shared

...I came across a story about a woman who tested positive for Covid and was so startled and terrified that she suffered a heart attack and didn't survive. Essentially, her cause of death was fear of the disease rather than the disease itself. Yet, it ended up being included in the total death count.

In contrast, members of the M4A subreddit underscored what they viewed as the seriousness of COVID-19. As one author asserted, “This is not a hoax; it's causing real harm by claiming lives and leaving numerous individuals with lasting damage.” Another author shared their reaction to encountering an unmasked family with 5 children: “It was frustrating to witness! Taking such a risk with the health of five kids—it's hard to believe they care about them at all.” A third user conveyed concerns about the transmission of the virus:

The thing is, all it takes is one major social gathering. From there, the spread can quickly reach each person's workplace, factories, food service, and so on. It's incredibly easy for things to spiral out of control rapidly.

**Figure 1:**
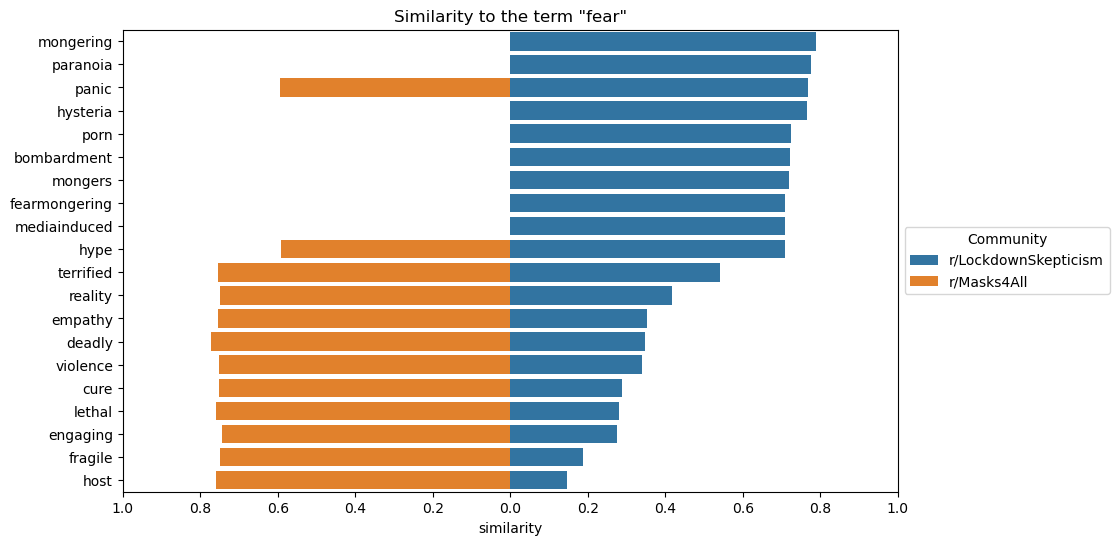
Word embedding results depicting terms similar to “fear” in LockdownSkepticism and Masks4All.

Word embeddings in our computational confirmation step, depicted in Figure 1, further underscored distinctions in conceptualizations of hazard severity across both subreddits. We identified terms for word embeddings based on emergent themes in our qualitative step. While the top 3 words similar to “fear” in LS were “mongering,” “paranoia,” and “panic,” the top 3 words associated with “fear” in M4A were “deadly,” “lethal,” and “host.” This distinction demonstrates divergent interpretations of “fear” in the context of COVID-19, with terms and arguments in one subreddit suggesting that the crisis was being overestimated, and terms and arguments in the other subreddit highlighting the urgency of the crisis.

In Figure 1, the 10 terms closest to the term “fear” based on cosine similarity for each community’s word embedding, sorted by closeness to the “r/LockdownSkepticism” community. The left side of the x-axis (orange) shows similarity to “r/Masks4All” and the right side (blue) shows similarity to “r/LockdownSkepticism.” Missing bars are terms that were used fewer than 10 times in that community.

#### Conceptualization of Social Responsibility

Many posts in the LS community argued for the importance of personal responsibility and individual freedom over collective obligations. One user shared:

When did I suddenly become accountable for others' health? It's never been the norm for everyone to be tasked with preserving the lives of complete strangers at any cost.

Likewise, another individual shared:

I personally dislike wearing masks myself. The argument is that masks help prevent your respiratory droplets from reaching others, so this pointless mandate is essentially about protecting others rather than yourself.

Unlike the LS subreddit, the M4A contributors emphasized how individuals’ actions impacted the health of others. One author shared:

I'm all for everyone having their rights, but personally, I choose not to exercise mine in a way that might harm others, even if it's technically allowed. [….] I’m not trying to mess with anyone’s freedoms; I just want to protect lives...

Another author shared concerns about the impact of others’ actions:

If there was a surefire way for me to stay completely protected while being out in public and doing my regular job, I’d simply shrug it off and move forward. Unfortunately, the choices others make end up affecting our health.

A third user elaborated on the potential for individuals’ actions to cause harm.

It’s unfortunate that those worsening the pandemic may not realize the full impact of their actions until it directly affects them or their loved ones. And even then, it’s unclear if they’ll fully comprehend it.

**Figure 2:**
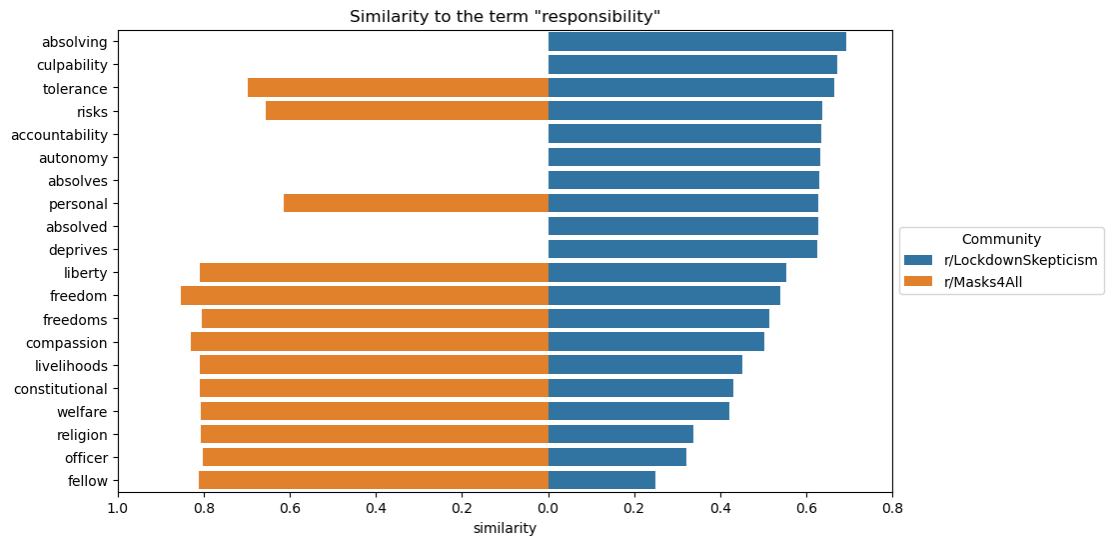
Word embedding results depicting terms similar to “responsibility” in LockdownSkepticism and Masks4All.

The word embeddings also demonstrated distinctions in conceptualizations of responsibility, as Figure 2 demonstrates. While “responsibility” was associated with “autonomy” and “personal” in LS, the same word was associated with “compassion” and “fellow” in M4A. This suggests that qualitative insights on differing perspectives toward social responsibility in both subreddits are representative of the discourse in each community, as one community espoused responsibility to the collective, while the other community stressed personal responsibility.

In Figure 2, the 10 terms closest to the term “responsibility” based on cosine similarity for each community’s word embedding, sorted by closeness to the “r/LockdownSkepticism” community. The left side of the x-axis (orange) shows similarity to “r/Masks4All” and the right side (blue) shows similarity to “r/LockdownSkepticism.” Missing bars are terms that were used fewer than 10 times in that community.

#### Conceptualization of Prevention Measures (Perspectives on Masking and Lockdown)

**Perspectives on Masking**

In the LS subreddit, individuals shared mixed perspectives on the efficacy of masking. For example, posts in our sample emphasized the possibility of user error and the importance of mask type. While some individuals expressed confidence in the efficacy of masks, others expressed concerns about incorrect usage or asserted that the types of masks being mandated were ineffective. As one individual described,

There has never been doubt about the effectiveness of N95 masks. The skepticism lies in the efficacy of makeshift single or two-layer cotton or cotton blend masks, raising questions about whether the potential benefits they offer outweigh the introduced risks. Additionally, there's consideration of whether they are more effective than simply adhering to proper cough and sneeze etiquette. Personally, I choose not to wear them.

However, authors in the M4A subreddit discussed the efficacy of different types of masks at length, promoted individual masking, and emphasized the need for government interventions in the form of mask mandates and lockdowns. As one author asserted,

A national mask mandate is necessary. Businesses should face fines of $10,000 for each instance where employees or patrons are maskless upon entry.” Some authors also shared that government interventions did not go far enough or were not adequately enforced.

As one author wrote, “This isn't a mask law; it's a joke.”

**Figure 3:**
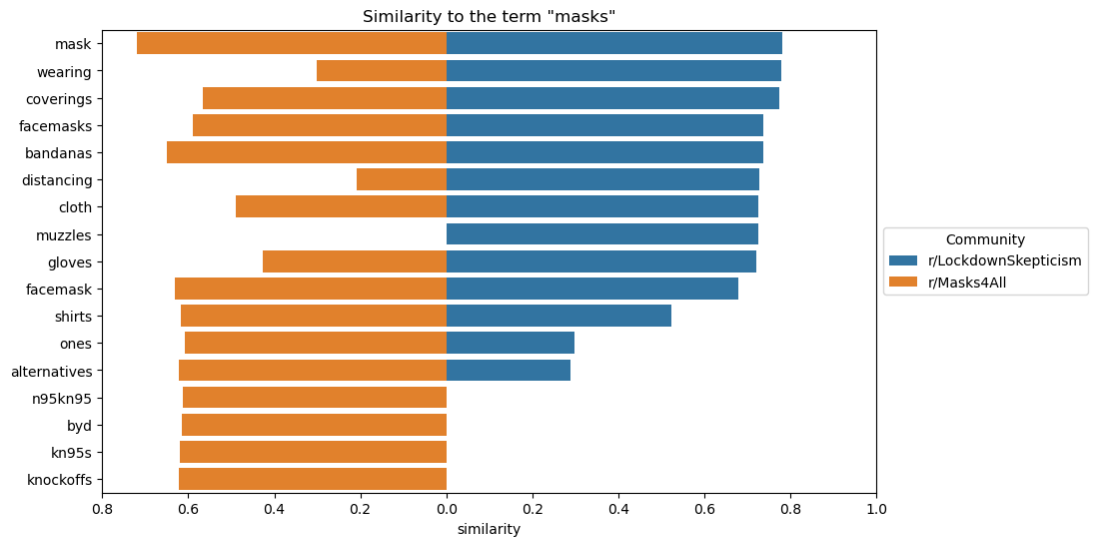
Word embedding results depicting terms similar to “masks” in LockdownSkepticism and Masks4All.

The word embedding analysis depicted in Figure 3 for LS captured terms broadly related to masks, such as “wearing,” “coverings,” and “bandanas.” However, the word embedding for M4A suggests a more dynamic discourse about types of masks, with “knockoffs,” “alternatives,” and “KN95s” being identified as similar to “masks.” Considered in tandem with our qualitative findings, this demonstrates that M4A was more involved in discourses around types of masks and their effectiveness at mitigating the threat of COVID-19.

The 10 terms closest to the term “masks” based on cosine similarity for each community’s word embedding, sorted by closeness to the “r/LockdownSkepticism” community. The left side of the x-axis (orange) shows similarity to “r/Masks4All” and the right side (blue) shows similarity to “r/LockdownSkepticism.” Missing bars are terms that were used fewer than 10 times in that community.

**Perspectives on Lockdown**

Members of the LS community asserted that individuals across the population had different levels of vulnerability to COVID-19, making a one-size-fits-all approach imperfect. As one member highlighted,

Care home residents clearly need more support than the general public, yet the only directive we receive is to 'stay home'. Resources … should be prioritized for care homes, while allowing others to make their own decisions about their actions and the associated risks.

LS community members also highlighted the harm that they believed the lockdown was causing members of the population, such as the elderly and children or youths. As one community member highlighted,

despite being active and independent, the quarantine in [my grandmother’s] assisted living home has led to a noticeable decline in her mental well-being, and she is now living in constant fear.

In this online community, several parents also expressed concerns about the impact of the lockdowns on the well-being and education of their children. In particular, authors emphasized the futility of distance learning and expressed a level of animosity toward teachers. As one author shared,

It happened to my child…The math test scores dropped significantly for my child, to the extent that they regressed by a whole grade!...All schools should be open. Education is crucial.

Authors in LS also emphasized the impact of lockdowns on both mental and physical health. As one individual described,

It's hard to determine what's worse these days: dealing with drug addiction or being addicted to fear-inducing content. Trying to reassure people that “it gets better” or “there's a lot to live for” is challenging when things don't seem that way. The prevailing atmosphere is one of uncertainty, unrest, anxiety, and overwhelming negativity that's almost impossible to escape. Many lives have been upended, changed, or ruined. Personally, as someone who has experienced and is currently battling severe depression again, it's difficult not to dwell on how bleak the future seems at this moment.

Members of this community also highlighted the perceived detrimental impact that government policies were having on individuals’ personal finances and the larger economy. As one post author shared,

Some people didn't want me to work… if I had stayed home, I would have already missed a car payment by now. I would have had to dip into my savings or accumulate credit card debt just to make ends meet. I've been saying this from the beginning. If anyone expects me to stay home, they should be willing to cover my bills and grocery expenses.

Meanwhile, in the M4A subreddit, contributors argued for the need for lockdowns. As one author shared,

If those in charge would simply unite and…genuinely enforce a lockdown… we could potentially make significant progress.

Another contributor shared a similar sentiment.

I'm ready to support Phase 1 lockdowns at this point. I'm willing to wear a mask, but I believe it's essential for people to stay home. That means closing schools, bars, restaurants, and gyms.

**Figure 4:**
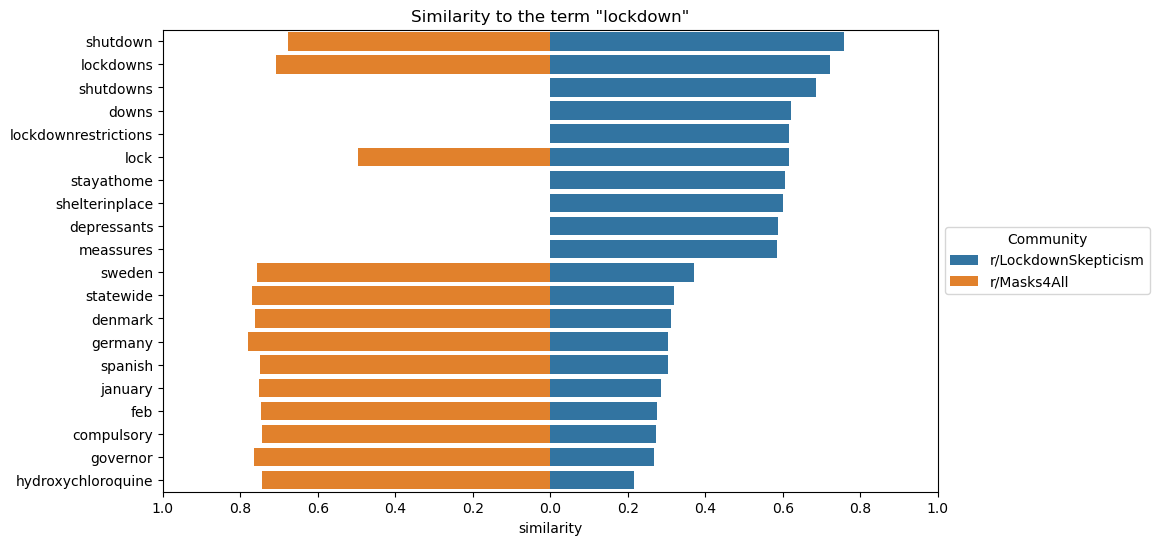
Word embedding results depicting terms similar to “lockdown” in LockdownSkepticism and Masks4All.

Word embeddings for LS and M4A reflect, as depicted in Figure 4, some of the distinct differences also captured by the qualitative analysis. Similarities of “Germany,” “statewide,” and “governor” to “lockdown” in the M4A subreddit may be due to the emphasis in this subreddit on the government’s responsibility to implement lockdowns.

The 10 terms closest to the term “lockdown” based on cosine similarity for each community’s word embedding, sorted by closeness to the “r/LockdownSkepticism” community. The left side of the x-axis (orange) shows similarity to “r/Masks4All” and the right side (blue) shows similarity to “r/LockdownSkepticism.” Missing bars are terms that were used fewer than 10 times in that community.

### Cocreating Group Identity

#### The LS Community

##### Affirming Collective Positions

Authors in this online community affirmed one another’s positions and asserted the reasonableness or apolitical nature of their viewpoints. As one author shared, “I think this subreddit is a rare space where people, regardless of their political affiliations, come together.” Authors also expressed the sentiment that they were in a “silent majority”. As one post stated: “Cancel culture has simply silenced people, pushing more individuals into the silent majority.”

##### Condemning the Outgroup

Authors in this subreddit also condemned the outgroup, referring to them as uninformed, privileged, antagonistic, or fear-mongering. The term “Doomer” was frequently used to refer to individuals who were perceived as overemphasizing the dangers of the pandemic, although this term was eventually banned by moderators. As one author shared,

A lot of people like to argue that those against the lockdown are selfish, ignoring the fact that a big reason why they might not care about it is that they haven't felt the negative consequences yet. It's pretty simple to preach about saving grandma when the lockdown doesn't have much impact on your own life.

#### The M4A Community

##### Social Media Advocacy

One of the ways in which members of the M4A community cocreated group identity was by commiserating with one another on advocacy fatigue. Sharing their own experiences advocating on Facebook, one author urged another to “keep fighting the good fight.”

##### Constructing the Outgroup as Threatening or Aggressive

Identity was also cocreated in this community as authors shared narratives about threatening or aggressive behavior against them by antimaskers. As one author shared,

It's genuinely surprising how the simple act of wearing masks has sparked such threatening and almost violent reactions in social situations.

##### Condemning the Outgroup

Authors also constructed group identity by condemning the outgroup. As one author shared

Yeah, I spend a good part of my day arguing with idiots who insist that wearing a mask deprives you of oxygen and kills brain cells. My comeback is usually pointing out that surgeons and medical professionals wear masks all the time during surgeries without any issues. If masks were as harmful as they claim, we'd see a lot of brain-damaged healthcare professionals.

## Discussion

### Principal Findings

This study examined how 2 ideologically distinct Reddit communities, M4A and LS, cocreated and communicated risk perceptions related to COVID-19. M4A supported COVID-19 mandates, especially masking, emphasizing the severity of the virus and the importance of collective risk-averse behaviors. Members favored government intervention to enforce protective measures. In contrast, LS acknowledged COVID-19 but questioned the necessity of preventive measures, citing minimal disease severity and concerns about the risks posed by these measures, such as economic harm, educational disruption, and social isolation. LS members prioritized individual responsibility and freedom over collective mandates. This divergence highlights differing risk perceptions shaped by group identity and values.

A major point of divergence between the 2 subreddits lies in their conceptualization of the severity of COVID-19. In LS, many contributors minimized the threat posed by the virus, portraying it as exaggerated by the media and government. This aligns with literature suggesting that individuals who are skeptical of authority tend to downplay risks that are emphasized by institutions [33]. LS contributors often referenced examples of fear-mongering, implying that public health narratives were designed to incite unnecessary panic. Such rhetoric reinforces findings from previous studies, which has linked lower perceived susceptibility to threats to disinclination from taking precautionary measures [34].

On the other hand, M4A contributors consistently emphasized the seriousness of COVID-19, often discussing the virus’s direct physical harms, including death and long-term health consequences [35]. This community portrayed COVID-19 as a significant public health crisis requiring urgent attention, which is consistent with research suggesting that individuals who trust medical experts and public health authorities are more likely to perceive the threat of COVID-19 as high [36]. Their framing of the pandemic aligns with other findings that demonstrate that clear communication from health authorities increases public trust and promotes adherence to health guidelines [37].

The word embeddings analysis further highlighted these divergent interpretations, with LS contributors associating “fear” with terms like “mongering” and “paranoia,” indicating skepticism toward the severity of the pandemic. In contrast, M4A contributors linked “fear” with “deadly” and “lethal,” emphasizing the real and immediate dangers of the virus. This distinction reflects the broader societal divide in COVID-19 risk perceptions, as described in other studies on ideological differences in health beliefs [34,38,39]. However, the LS contributors did not just downplay the risks of COVID-19; they also focused on alternative risks, such as risks to education and the economy.

The 2 subreddits also diverged in their conceptualizations of social responsibility. In LS, contributors argued for personal autonomy and individual responsibility over collective obligations. This supports other studies that suggest that individuals who prioritize personal freedom are more likely to resist collective measures such as mask mandates [40]. Many in the LS subreddit saw government interventions as an infringement on personal liberties, a theme that has been widely observed in other studies of antilockdown protests [40,41].

In contrast, M4A contributors emphasized the importance of protecting others, portraying mask-wearing and social distancing as moral imperatives to safeguard public health. Their focus on collective responsibility resonates with findings from research on prosocial behavior during pandemics, which show that individuals with higher levels of empathy and concern for others are more likely to engage in protective behaviors [42].

The word embeddings analysis reinforced these qualitative insights. In LS, “responsibility” was associated with terms like “autonomy” and “personal,” highlighting an individualistic approach to social responsibility. In contrast, M4A linked “responsibility” with “compassion” and “fellow,” indicating a collective approach that prioritizes the well-being of others. These findings are consistent with research suggesting that political and social ideologies shape how people interpret their obligations to others during public health crises [37,43,44].

The subreddits' discussions on prevention measures, such as masking and lockdowns, further illustrate their divergent ideologies. LS contributors were generally skeptical of mask mandates, focusing on the perceived inefficacy of certain types of masks, such as cloth face coverings, and questioning whether government-imposed lockdowns were justified given the collateral damage to mental health and the economy. This echoes existing evidence showing findings that resistance to public health interventions often arises from a perceived conflict between individual freedoms and collective safety [45].

In contrast, M4A contributors advocated for strict enforcement of mask mandates and lockdowns, arguing that these measures were essential to control the virus's spread. Their support for government intervention is consistent with research showing that individuals who trust government institutions are more likely to endorse stringent public health measures [46]. Contributors also expressed frustration with the perceived lack of enforcement, reflecting broader concerns about the efficacy of public health measures when compliance is inconsistent [37,47].

The word embeddings analysis revealed differences in how each community discussed prevention measures. In LS, terms like “bandanas” and “coverings” were associated with masks, suggesting a more critical view of their efficacy, particularly with regard to non–medical-grade masks. In M4A, the word embedding highlighted a more detailed discourse around mask types, including “KN95s” and “alternatives,” reflecting a more nuanced interest in which masks offer the best protection. This aligns with previous research on online communities’ deliberation about masks [48].

Both subreddits demonstrated strong in-group cohesion and outgroup condemnation, a hallmark of online communities where social identity is coconstructed and reinforced [49,50]. In LS, contributors framed their community as part of a “silent majority,” united by their skepticism of government overreach and disdain for what they perceived as the “doom mongering” of prolockdown advocates. This reflects findings from SIT, which suggests that individuals derive a sense of belonging from shared beliefs and norms within their group [51,52].

Similarly, M4A contributors bonded over their shared advocacy for mask-wearing and public health measures, often commiserating over the challenges of persuading others to adopt protective behaviors. This aligns with research that suggests advocacy communities reinforce group identity by emphasizing collective goals and shared experiences [51]. Both subreddits engaged in outgroup condemnation, with LS contributors accusing prolockdown advocates of overreacting and M4A contributors portraying antimaskers as reckless and irresponsible. This pattern of polarization is consistent with research on intergroup conflict, which shows that opposing groups often define themselves in contrast to their perceived opponents [53].

### Implications

This study proffers several implications for public health, particularly in shaping targeted health messaging, understanding risk perception through social identity, and leveraging online communities for health engagement. The findings highlight how group norms and social identity influence risk perception, with important implications for health behavior. In addition, the study underscores the role of outgroup condemnation in public health discourse and provides valuable insights for policymakers seeking to design more effective and inclusive health interventions.

Findings from this study demonstrate that individuals within ideological communities tend to share common perceptions of risk, shaped by their identity group. Public health strategies can benefit from recognizing and being responsive to these group dynamics because understanding how these different communities perceive risk is essential for crafting effective health communication strategies. Public health practitioners should tailor messaging to align with the specific concerns and values of audience segments, rather than adopting a one-size-fits-all approach. For example, members of the M4A subreddit are more likely to respond positively to messages emphasizing the collective benefits of preventive measures like masking. As such, messaging for communities who share this ideology may be framed around community protection, solidarity, and efforts to reduce the strain on the health care system. In contrast, members of the LS community may be more responsive to messaging that acknowledges their concerns about the potential harms of such measures, while still emphasizing personal responsibility for health outcomes. Messaging for communities who share this ideology could focus on individual autonomy in making informed health choices while highlighting the benefits of preventive measures in protecting personal freedoms, by reducing long-term government interventions. Developing parallel but distinct messaging strategies for differing ideological groups may improve engagement and compliance with public health recommendations.

Another effective strategy to leverage these dynamics is by identifying and collaborating with trusted individuals within these online communities. For instance, engaging with influencers and thought leaders who have credibility within ideological groups can increase the reach and acceptance of public health messages. Rather than relying solely on traditional health institutions, which communities may distrust, partnerships with community leaders or digital content creators could enhance the effectiveness of outreach efforts.

Given the increasing influence of online communities, public health agencies could prioritize actively monitoring and engaging with these online communities to better understand public concerns, misinformation, and nonadherence to health recommendations. Beyond passive monitoring, public health agencies should consider creating an official presence within these spaces to directly address concerns, counter misinformation in real time, and foster constructive dialogue. In addition, collaborating with moderators of online communities to host question and answer sessions or ask me anything with medical professionals could help dispel misconceptions and humanize public health efforts.

The LS community’s concerns about the mental health and social implications of lockdowns and masking mandates highlight a critical area for further public health intervention. While pandemic measures provide clear physical health benefits, they also carry psychological and economic costs. A holistic approach to public health policy making should carefully weigh these trade-offs, ensuring that both the physical and mental well-being of communities are considered. One potential approach is integrating mental health support into public health initiatives, such as pairing pandemic or public health efforts with resources for coping strategies, virtual counseling, or community-based peer support groups. In addition, transparent and empathetic communication about these trade-offs may help in building public trust and compliance, particularly if public health policies are framed as adaptable rather than rigid, acknowledging evolving circumstances and community feedback.

SIT suggests that group norms strongly influence individual behavior, particularly in the context of health decision-making [54]. Adherence to group norms reinforces a sense of shared identity, shaping responses to public health measures. This is evident in the LS community’s emphasis on individual freedoms compared to M4A’s focus on collective responsibility. The study highlights how online communities serve as spaces where these group identities are both formed and reinforced, with significant implications for public health engagement strategies. Encouraging positive norm-setting within these communities—such as promoting testimonials from individuals within the group who successfully adopted public health measures—could be a way to shift attitudes in a culturally and ideologically resonant manner.

Moreover, findings from this study reveal how ingroup-outgroup dynamics shape public health discourse. Members of both subreddits reinforced their identities by condemning the opposing groups, aligning with existing research suggesting that intergroup interactions may become contentious when there is divergence in group goals and objectives [53,55]. As such, public health professionals could be mindful of these dynamics when designing health campaigns that could potentially deepen intergroup divergence. A potential strategy for addressing this could be framing public health messages around shared values that transcend ideological divisions. For instance, instead of emphasizing compliance with mandates, messaging could highlight common concerns such as economic stability, family well-being, or the protection of vulnerable loved ones. In addition, using framing more specific to local communities or demographics may resonate more effectively, as opposed to broad national directives.

Our findings align with emerging international research on COVID-19 risk perceptions and online group identity. For example, a study conducted in South Korea found that social media was a key space for amplifying risk perceptions, with ideological communities shaping public discourse around health risks and protective behaviors [56]. Similarly, global analyses of COVID-19 misinformation and social fragmentation online have shown that different social media platforms (eg, X [formerly known as Twitter], Facebook [Meta], and Instagram [Meta]), can reinforce ideological silos that influence how users assess health risks [57]. While our study focused on Reddit communities in the United States, these patterns mirror findings from international contexts, suggesting a broader transnational phenomenon of ideologically driven risk perception across digital platforms [57].

### Limitations

This study is not without limitations. First, there are limitations pertaining to the sample populations. The study focused on 2 specific online communities on Reddit, M4A and LS, which may differ from other online or offline communities. The beliefs and risk perceptions identified in these groups may be distinct from the viewpoints prevalent in other Reddit communities. Future studies should adopt a broader scope for a deeper understanding of risk conceptualizations in digital spaces. In addition, the sample was self-selecting, meaning that only individuals who were already actively engaging with these communities were included, potentially skewing the data toward more extreme views on both ends of the spectrum, while overlooking more moderate perspectives.

The study assumes that the 2 communities, M4A and LS, are relatively homogeneous in their risk perceptions. However, there could be significant variation within each group, with members holding more nuanced or even contradictory views. The study might not fully capture this internal diversity, leading to the overgeneralization of group attitudes.

The study focused exclusively on Reddit communities, which may not capture the full range of public discourse on COVID-19 risks across other platforms such as Twitter, Facebook, or Instagram. Different social media platforms may foster different types of engagement, and the dynamics of risk cocreation could vary significantly depending on the platform’s technological affordances, user base, and interaction styles. More so, the discussions analyzed were specific to a certain period during the COVID-19 pandemic, and risk perceptions may have evolved over time as new information emerged, or as individuals’ experiences with the pandemic changed. Consequently, the findings do not fully capture the shifts in public sentiment and behavior as the pandemic progressed. Without a longitudinal analysis, it's difficult to assess how group norms and risk perceptions within these communities might change over time, especially in response to new developments or policy changes related to the pandemic. Future studies should use a longitudinal approach to analyze the evolution of risk perception over time across communities.

Finally, the study relied on content analysis of online discussions, which may miss important nonverbal cues, such as tone or body language, that could provide further insight into how risk perceptions are expressed and reinforced. In addition, users' comments in online forums may not always reflect their true beliefs or behaviors, as individuals might adopt different personas or engage in “performative” behavior when interacting with others online.

### Future Research Directions

This study lays the groundwork for additional research into how online group dynamics and social identity influence health behaviors beyond the context of COVID-19. Future studies could explore how these findings apply to other health crises, such as vaccine uptake, climate change, or noncommunicable diseases. In addition, researchers should investigate how interventions that target group identity could be designed to shift risk perceptions and encourage more protective health behaviors across different ideological communities. In doing so, we can develop more nuanced public health strategies that resonate with diverse populations, ultimately fostering more robust collective responses to global health challenges. While this study focuses on 2 US-based Reddit communities, similar dynamics have been observed in countries such as South Korea where ideological divides mediate engagement with health information [56]. Future research should continue to explore these dynamics across geographic and cultural contexts to better inform targeted public health communication strategies in both local and global digital environments.

### Conclusions

This study has used 2 divergent Reddit communities to understand the cocreation of risk meanings through social learning and the role of group identity in the development and communication of risk meanings online. Findings from this study are contributing to existing literature on the SIT and SARF, highlighting how group memberships inform norms, attitudes, and perceptions within a social context.

This study highlights the role of online communities in shaping perceptions of risk and health behavior, particularly within ideologically distinct groups like M4A and LS. By exploring how these communities construct and communicate risks related to COVID-19, it becomes evident that social identity and group norms play a significant role in how individuals approach public health measures. This study has demonstrated that divergent risk perceptions are not merely based on the nature of the risk but are deeply influenced by group dynamics, social identity, and collective belief systems.

The findings from this study underscore the importance of tailoring public health messaging to the values and concerns of specific groups, rather than relying on uniform strategies. Public health practitioners can leverage insights from online communities to better understand public concerns, target messaging more effectively, and address resistance to health interventions. In addition, this research adds to the growing body of literature on how group identity shapes risk perception, providing a foundation for developing interventions that align with the norms of different social groups.

## Appendix

Multimedia Appendix 1 Topics.

## Abbreviations

LDA: latent Dirichlet allocation
LS: LockdownSkepticism
M4A: Masks4All
RQ: research question
SARF: Social Amplification of Risk Framework
SIT: Social Identity Theory

## Data Availability

The datasets generated or analyzed during this study are available in the Dataverse repository [58].
