# Supplementary material for: Exploring Conceptualizations of COVID-19 Risk in Ideologically Distinct Online Communities: A Computational Grounded Theory Analysis
Source: J Med Internet Res. 2025 Jun 24;27:e67968. doi: 10.2196/67968 (PMC12238777; doi:10.2196/67968)
Supplement: Multimedia Appendix 1 [file jmir_v27i1e67968_app1.docx]

| Topic | Summary |
| --- | --- |
| Relationships | Impact of COVID-19 on relationships |
| Covid protective measures | Discussion about COVID-19 protective measures |
| Mask | Discussion about masks |
| Education | Impact of COVID-19 on education |
| Controversies/political | Discussion of controversies and policies around COVID-19 |
| Information | Discussion of information sources related to COVID-19 |
| Credibility | Discussion of the credibility of information |
| Regulations | Discussion of beliefs around regulations |
| Symptoms/severity | Discussion about COVI-19 symptoms and severity |
| Lockdown | Discussion about lockdowns |
